# The Role of Mesothelin in Activation of Portal Fibroblasts in Cholestatic Liver Injury

**DOI:** 10.3390/biology11111589

**Published:** 2022-10-28

**Authors:** Takahiro Nishio, Yukinori Koyama, Hiroaki Fuji, Kei Ishizuka, Keiko Iwaisako, Kojiro Taura, Etsuro Hatano, David A. Brenner, Tatiana Kisseleva

**Affiliations:** 1 Department of Medicine, University of California San Diego, 9500 Gilman Drive, #0063, La Jolla, CA 92093, USA; 2 Department of Surgery, University of California San Diego, 9500 Gilman Drive, #0063, La Jolla, CA 92093, USA; 3 Department of Surgery, Graduate School of Medicine, Kyoto University, 54 Kawaharacho Shogoin, Sakyo-ku, Kyoto 606-8507, Japan; 4 Department of Medical Life Systems, Faculty of Life and Medical Sciences, Doshisha University, 1-3 Tataramiyakodani, Kyotanabe 610-0394, Japan; 5 Department of Gastroenterological Surgery and Oncology, Kitano Hospital Medical Research Institute, 2-4-20 Ogimachi, Kita-ku, Osaka 530-8480, Japan

**Keywords:** cholestatic fibrosis, activated Hepatic Stellate Cells, activated Portal Fibroblasts

## Abstract

**Simple Summary:**

Fibrosis is a common response to chronic tissue injury and plays a critical role in many diseases across medical subspecialties. To date, there are few therapies with limited effectiveness to treat fibrotic diseases. Expression of mesothelin was detected in activated Portal Fibroblasts, which are the major contributors to cholestatic liver fibrotic diseases such as primary and secondary biliary cholangitis and primary sclerosing cholangitis. This manuscript summarizes our most recent findings of the role of mesothelin in the pathogenesis of cholestatic fibrosis, and as a common mediator of tissue fibrosis. The function of mesothelin was linked to the activation of TGFβ1-mediated fibrogenic responses and FGF-induced proliferation of tissue fibroblasts.

**Abstract:**

Fibrosis is a common consequence of abnormal wound healing, which is characterized by infiltration of myofibroblasts and formation of fibrous scar. In liver fibrosis, activated Hepatic Stellate Cells (aHSCs) and activated Portal Fibroblasts (aPFs) are the major contributors to the origin of hepatic myofibroblasts. aPFs are significantly involved in the pathogenesis of cholestatic fibrosis, suggesting that aPFs may be a primary target for anti-fibrotic therapy in cholestatic injury. aPFs are distinguishable from aHSCs by specific markers including mesothelin (Msln), Mucin 16 (Muc16), and Thymus cell antigen 1 (Thy1, CD90) as well as fibulin 2, elastin, Gremlin 1, ecto-ATPase nucleoside triphosphate diphosphohydrolase 2. Msln plays a critical role in activation of PFs, via formation of Msln-Muc16-Thy1 complex that regulates TGFβ1/TGFβRI-mediated fibrogenic signaling. The opposing pro- and anti-fibrogenic effects of Msln and Thy1 are key components of the TGFβ1-induced activation pathway in aPFs. In addition, aPFs and activated lung and kidney fibroblasts share similarities across different organs with expression of common markers and activation cascade including Msln-Thy1 interaction. Here, we summarize the potential function of Msln in activation of PFs and development of cholestatic fibrosis, offering a novel perspective for anti-fibrotic therapy targeting Msln.

## 1. Introduction

Fibrosis is a common consequence of abnormal wound healing, which is characterized by infiltration of myofibroblasts and formation of fibrous scar, eventually leading to loss of organ function. Myofibroblasts, which are absent from normal tissue and appear de novo in response to tissue injury, play a crucial role during physiological tissue repair [1]. Myofibroblasts are transiently activated to restore the integrity of the injured tissue by producing extracellular matrix (ECM) that is followed by remodeling and resolution in the final phase of repair [2,3]. In contrast, chronic or repeated injury in any tissue may cause dysregulation of the repair process with persistent myofibroblast activation, resulting in excessive accumulation of ECM and scar formation [4]. Determining the potential source of myofibroblasts and the mediators of their activation pathway is crucial for revealing effective targets for antifibrotic therapy in fibrotic diseases.

In liver fibrosis, the proposed sources of myofibroblasts are Hepatic Stellate Cells (HSCs), Portal Fibroblasts (PFs), bone-marrow derived fibrocytes, epithelial cells, or mesenchymal stromal cells [1,5,6,7]. Among these cell types, activated HSCs (aHSCs) and activated PFs (aPFs) are the major contributors to the origin of hepatic myofibroblasts [5,8]. HSCs have been well characterized as the pericytes unique to the liver [9], which reside in the space of Disse, store vitamin A, and become activated in response to a variety of hepatotoxic injury, including chronic hepatitis B or C virus infection, alcohol-associated liver disease, and nonalcoholic steatohepatitis (NASH) [4]. On the other hand, PFs comprise a small population of cells that surround the biliary component, serving to maintain the physical integrity of the portal tract [6,10]. PFs become activated and proliferate around the portal area, responding to biliary obstruction and damage. Accordingly, aPFs are a major source of type 1 collagen-producing myofibroblasts in cholestatic liver fibrosis, including biliary atresia, primary biliary cholangitis (PBC), secondary biliary cholangitis (SBC), or primary sclerosing cholangitis (PSC) [8], whereas they show little contribution to hepatotoxic liver fibrosis such as viral hepatitis, alcohol-associated hepatitis and NASH.

The contribution of aPFs to liver fibrosis of different etiologies has been poorly understood, mainly because of the difficulties in isolating PFs and myofibroblasts. Several studies in humans and experimental models of cholestatic fibrosis in mice indicated that aPFs are significantly involved in the pathogenesis of cholestatic fibrosis, suggesting that aPFs may be a primary target for anti-fibrotic therapy in cholestatic injury [6,10]. The methods for cell isolation are based on enzymatic digestion and the outgrowth from enriched non-parenchymal fraction [11,12,13], and development of flow cytometry-based sort purification techniques [8] have revealed potential markers of aPFs, which include Thy1, fibulin 2, elastin, Gremlin 1, ecto-ATPase nucleoside triphosphate diphosphohydrolase 2 (NTPD2), mesothelin (Msln), and mucin 16 (Muc16)(9, 14). Notably, recent research has demonstrated that Msln-Muc16 (CA125, ligand of Msln)-Thy1 complex is crucially involved in the activation and proliferation of PFs via the transforming growth factor-β (TGFβ)-mediated profibrogenic cascade in response to cholestatic liver injury [14]. Thus, Msln is identified as a significant marker of aPFs and a potential target of antifibrotic therapy in cholestatic fibrosis [15]. 

Additionally, Msln is implicated as a common marker of fibroblast and smooth muscle cell precursors across organs including lung and kidney as well as liver [16]. Based on the functional link between Msln expression and profibrogenic pathway in aPFs, it was indicated that tissue fibroblasts and activated myofibroblasts in different organs have shared characteristics, and the Msln-Thy1-mediated signaling is universally associated with the progression of fibrosis via activation of tissue fibroblasts in the lung and kidney [17]. Msln may be a key mediator of TGFβ1-inducible activation in the tissue fibroblasts across organs, providing insight into a therapeutic strategy for treatment of parenchymal organ fibrosis. This review will summarize the potential role of Msln in activation of fibroblasts, highlighting the biological behaviors of aPFs in cholestatic fibrosis; and will offer a perspective of novel strategies for anti-fibrotic therapy targeting Msln.

## 2. Origin of Hepatic Myofibroblasts

Activated myofibroblasts emerge, proliferate, and produce excessive ECM as a common consequence of chronic injury regardless of tissues and organs. A variety of cell types have been proposed as the source of myofibroblasts depending on the type of tissue and injury, which may include pericytes, resident fibroblasts, bone-marrow derived fibrocytes, and transition from epithelial or endothelial cells [6,18,19]. Using the experimental model of reporter Col-GFP mice (in which Collagen-α1(I) (Col1α1) promoter drives expression of the GFP reporter gene in real time) [20], activated myofibroblasts can be adequately detected as Col-GFP^+^ cells infiltrating into injured tissues, yet they are heterogeneous in origin and need further characterization. In the cholestatic liver, PFs and HSCs are the major source of type I collagen-producing myofibroblasts, while the contribution of fibrocytes is minimally observed (<4% of total myofibroblasts) s [8,14,21]. 

The composition of hepatic myofibroblasts in fibrotic liver remains a subject of discussion [9,14]. Electron microscopy and immunohistochemistry has provided evidence that both portal fibroblasts and hepatic stellate cells can transform into myofibroblasts [22,23,24]. Cell fate mapping have demonstrated activated HSCs and aPFs are the major sources of myofibroblasts in experimental models of liver fibrosis [14,25,26]. However, the composition of myofibroblasts may vary substantially, depending on the etiology of hepatic fibrosis. HSCs are primarily activated in response to toxic liver injury, which mostly affects the centrilobular and perisinusoidal regions in the liver [27], whereas both aPFs and HSCs contribute to cholestatic liver fibrosis that results mostly from periportal injury [8,21]. Specifically, activated portal fibroblasts comprise 70% of myofibroblasts at the onset of experimental cholestatic injury (5 days after BDL), whereas HSCs are increasingly activated with the progression of cholestatic injury (17 days after BDL), eventually constituting the largest myofibroblast population (20 days after BDL) [8,21]. The composition of the fibrous scar can also vary between toxic and cholestatic fibrosis. Thus, increased deposition of elastin fibers is more common in cholestatic fibrosis, since aPFs are the major source of elastin. Meanwhile, Collagen Type I, composed by a triple helix of collagen-1α(1) and collagen-1α(2), is the most abandon component of ECM in fibrous scar in the liver independent of the etiology. 

### 2.1. Hepatic Stellate Cells

**Figure 1:**
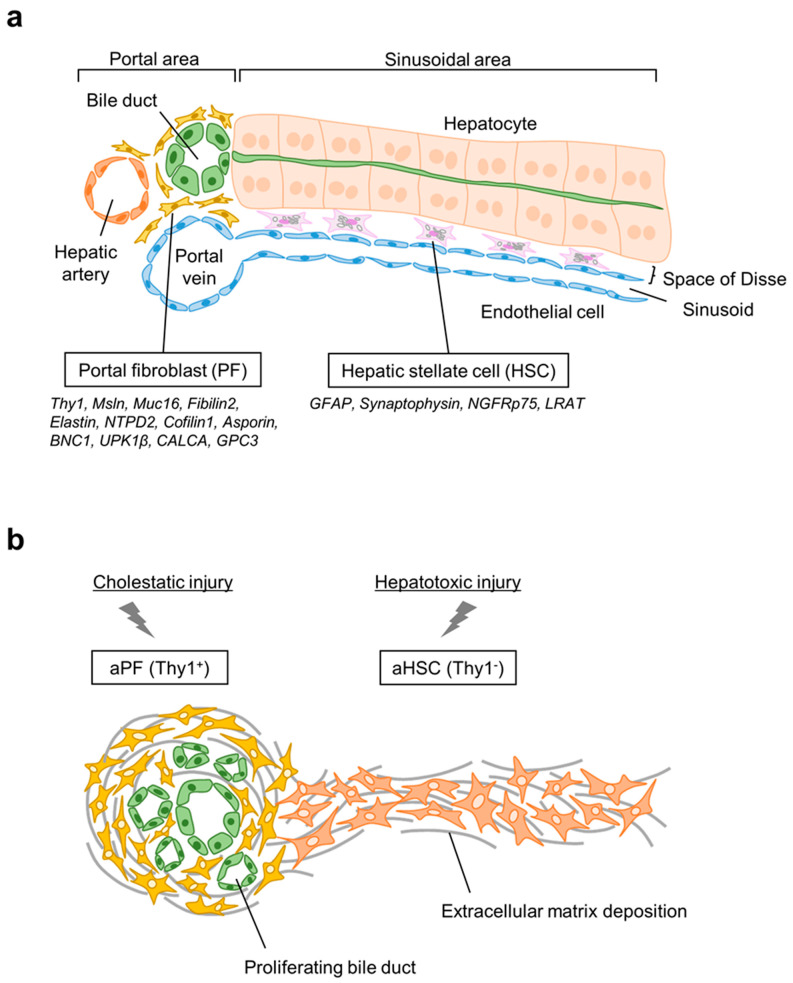
Contribution of PFs and HSCs to hepatic myofibroblasts in response to liver injury. (**a**) PFs are located surrounding portal area, while HSCs are located in the space of Disse, which is a gap between sinusoidal endothelial cells and hepatocytes cluster. (**b**) PFs are primarily activated in response to cholestatic injury giving rise to Thy1^+^ aPFs/myofibroblasts which proliferate around portal area and form biliary fibrosis. HSCs are predominantly activated in response to hepatotoxic injury, and aHSCs infiltrate along the sinusoidal area and proliferate into parenchymal region forming a bridging fibrous scar.

Under physiological conditions, HSCs serve as liver pericytes residing in the space of Disse, and are the major storage site of vitamin A, exhibiting a quiescent phenotype [28,29]. Quiescent HSCs express desmin and neuronal markers, including glial fibrillary acidic protein (GFAP), synaptophysin, nerve growth factor receptor (NGFR) p75, and lecithin-retinol acyltransferase (LRAT) [4,9,30,31] (Figure 1a). Due to the function of HSCs as pericytes and their close association with endothelial cells and hepatocytes, qHSCs are predominantly activated and proliferate in response to toxic liver injury as shown in experimental models (CCl_4_, NASH, viral hepatitis, alcohol-associated liver injury) and in patients [10,32,33](Figure 1b). On activation, qHSCs decrease vitamin A lipid droplets and downregulate GFAP and peroxisome proliferator-activated receptor–γ (PPARγ), leading to differentiation into collagen type I producing myofibroblasts with upregulation of α-smooth muscle actin [34,35]. TGFβ1 signaling is the major profibrogenic cascade that derives HSC activation via a SMAD2-dependent or SMAD3-dependent manner [36]. Additionally, connective tissue growth factor (CTGF) and IL-13 facilitate TGFβ1-independent activation of HSCs and secretion of Collagen Type I [37]. It has been reported that HSCs are composed of heterogeneous clusters, which include the classic fibrogenic myofibroblast cluster, proliferating cluster, intermediately activated cluster, and immune and inflammatory cluster [33]. Recently, it was suggested that a unique proliferating cluster of HSCs contribute to promote liver regeneration following partial hepatectomy in mice by induction of hepatocyte hypertrophy as a potential role of liver pericytes [38]. 

### 2.2. Portal Fibroblasts

PFs are distinctively identified as liver resident fibroblasts surrounding the biliary component, which comprise a population of 0.1% of total liver cells and physiologically maintain the integrity of the portal tract [6,10] (Figure 1a). They were first described as mesenchymal cells distinct from sinusoidal endothelial cells, and thereafter PFs were identified as periductular fibroblasts or portal/periportal mesenchymal cells [6,39]. Like aHSCs, aPFs can give rise to activated myofibroblasts that drive hepatic fibrosis. Potentially owing to the specific localization surrounding the biliary tract, PFs get activated in response to bile duct damage caused by biliary obstruction and cholestasis. aPFs proliferate around biliary components and crucially contribute to “onion skin-like” periductal fibrosis [40]. It remains unknown if aPFs can be “inactivated” during regression of cholestatic fibrosis. aPFs are distinguishable from aHSCs by the expression of Thy1 [41,42,43], fibulin 2 [41], elastin [44], NTPD2 [45], cofilin 1 [46], Msln, Muc16, asporin, basonuclin 1 (BNC1), uroplakin-β (UPK1β), calcitonin- related polypeptide α (CALCA), glipican 3 (GPC3)(Figure 1b) [8,14]. Recently, functional molecular interaction among Msln, Muc16, and Thy1 in the regulation of fibrogenic effect of aPFs has been demonstrated, offering a novel target of anti-fibrotic therapy in cholestatic liver fibrosis, such as PBC, SBC, and PSC [14,17]. 

## 3. Activation of HSCs in Response to Cholestatic Injury

When the gene expression profile of BDL-activated PFs was compared with aHSCs activated in response to cholestatic (BDL) or toxic (carbon tetrachloride, CCl_4_) injury [8], aPFs exhibited a myofibroblast-like phenotype, sharing mRNA expression of fibrogenic genes commonly expressed in BDL- and CCl_4_-activated aHSCs [47]. These genes included Col1a1, Col1a2, Col2a1, TIMP-1, Spp1, TGFβ-RI, and Vimentin, and were expressed in aPFs to a level comparable to BDL- and CCl_4_-aHSCs. Furthermore, aPFs upregulated expression of IL-18R, IL-25R, and other genes that distinguish them from aHSCs. Interestingly, BDL-aHSCs shared more similarity with aPFs than with CCl_4_-aHSCs [8], suggesting that aHSCs may mimic the phenotype of aPFs in response to cholestatic liver injury. The highest expression of αSMA was detected in CCl_4_-aHSCs, while expression of αSMA was much lower in aPFs expressed [47].

In comparison with BDL-activated aPFs, aPFs comprised only to 12–13% of Collagen Type I expression cells in response to CCl_4_ injury. Unlike BDL-aPFs and CCl_4_-aHSCs, CCl_4_-aPFs did not exhibit a fully activated phenotype and minimally contributed to toxic liver fibrosis [8,48]. 

Further analysis of the gene expression profiles of the BDL-activated aPFs revealed upregulation of biological pathways linked to biological adhesion, response to stimulus, developmental process and cellular organization, locomotion, focal adhesion, cell adhesion molecules, regulation of actin cytoskeleton, induction of the profibrogenic Wnt signaling pathway [8]. aPFs responded to IL-25 stimulation by induction of IL-13 [47]. Although IL-13 is implicated in HSC activation, and IL-13 levels are up-regulated in patients with liver cirrhosis [37,49], the role of IL-13 in cholestatic liver injury has not been well defined. Studies suggested that IL-25–mediated production of IL-13 by BDL-aPFs may stimulate activation of HSCs. Upon stimulation with IL-13, aHSCs increased production of CTCF and upregulated Co1la1, aSMA, TIMP1, and mRNA, suggesting that aPFs may locally facilitate HSC activation via production of IL-13 [8]. A more detailed analysis demonstrated that stimulation of HSCs with IL-13 causes up-regulation of IL-13Ra2 expression (but not IL-13Ra1 or IL-4) and transcription of IL-13 target genes such as Tenascin C and Eotaxin. IL-13-mediated HSC activation was attributed to activation of ERK1/2 [8,47,50]. In turn, stimulated aHSCs did not produce IL-13.

## 4. Activated Portal Fibroblasts in Cholestatic Fibrosis

Chronic cholestatic injury induces hepatocyte apoptosis, ductular proliferation, inflammation, and activation of myofibroblasts [51], resulting in cholestatic fibrosis which is characterized by ECM scar formation in the periportal area [52]. Both aPFs and aHSCs can be a potential source of hepatic myofibroblasts that drive cholestatic fibrosis [8,21]. Although the origin and contribution of myofibroblasts to cholestatic fibrosis remains controversial, studies in humans and experimental models have implicated aPFs in the pathogenesis of cholestatic fibrosis. Cholestatic injury primarily acts on PFs to proliferate and differentiate into type I collagen-producing myofibroblasts [6,53]. Using the reporter Col-GFP mice, aPFs were shown to comprise 70% of myofibroblasts at the onset of cholestatic fibrosis in the experimental model of bile duct ligation (BDL) [8,14]. The multidrug resistance gene 2 knockout (Mdr2^−/−^, also known as Abcb4^−/−^) mouse is another well-established model of chronic cholestatic liver injury. Deficiency of Mdr2, a canalicular phospholipid flippase, disrupts biliary phospholipid secretion and increases toxic bile acid, leading to peri-cholangitis and periductal fibrosis which resembles pathology of PSC [40,54,55,56,57,58,59]. Thy1 expressing aPFs significantly contribute to the Col-GFP^+^ hepatic myofibroblasts in Mdr2^−/−^ mouse during the progression of cholestatic fibrosis, and it is indicated that aPFs can serve as a target of antifibrotic therapy in cholestatic injury [17,21]. In human livers of PSC patients, expression of MSLN and THY1 are upregulated, showing a correlation with the stage of liver fibrosis. In support, human MSLN^+^THY1^+^αSMA^+^ aPFs isolated from graft livers with cholestasis which were declined for transplantation were shown to express aPF-specific markers UPK1b, CD200, EMILIN2, BNC1, ASPN, GPC3, and GREM1, similar to that observed in mouse aPFs, suggesting MSLN-expressing aPFs are significant contributors to human cholestatic fibrosis and a potential target of anti-fibrotic therapy [17]. 

## 5. Biological Function of Mesothelin, Muc16, and Thy1

### 5.1. Mesothelin

Msln, a glycosylphosphatidylinositol (GPI)-anchored membrane protein, is a surface marker expressed on mesothelial cells [60,61]. The biological functions of Msln have been poorly understood because Msln-deficient mice do not show a detectable phenotype under physiological conditions [62]. On the other hand, Msln is known to be highly expressed in several human tumors including mesothelioma, ovarian cancer, pancreatic adenocarcinoma, lung adenocarcinoma, and cholangiocyte carcinoma [63,64,65], and thus it has attracted attention as a potential target for anti-cancer therapy [61,66] by newly developed strategies of immunotherapy using recombinant immunotoxin, antibody-drug conjugates, chimeric monoclonal antibody, and chimeric antigen receptor T cell therapy [67,68,69,70,71,72,73,74]. Msln expression is abundant in normal mesothelial cells, which are major components of the mesothelial layer lining parenchymal organs and serosal cavities [62]. Notably, lineage tracing approach using genetic labeling of Msln^+^ cells during the embryonic development (using MslnCre/ERT mice crossed to the reporter Rosa26-flox-Stop-flox-GFP mice) demonstrated that Msln expression was observed in fibroblast precursors in the liver, lung, and kidney, presenting a mesenchymal signature with surface phenotype including Thy1^high^, CD34^high^ CD44^low^ CD105^low^ [16]. The potential expression of Msln in tissue fibroblasts may be linked to the molecular mechanism of their activation via the TGFβ1-inducible profibrogenic pathway, which has been determined by the role of aPFs in cholestatic liver fibrosis [14].

### 5.2. Mucin 16 (CA125)

Muc16 is the murine analogue of human CA125 [75]. CA125 is a member of the membrane-tethered family of mucins, which contains a transmembrane domain with a short cytoplasmic domain, and highly glycosylated at N-terminus [66]. Studies of human ovarian cancer have revealed that the cancer antigen CA125 can serve as a ligand of MSLN [76,77], and co-expression of MUC16 and MSLN is known to be associated with the invasion process of cancers [78,79,80,81], with MUC16 promoting the potential role of MSLN in tumor adhesion, and metastasis [76,82]. CA125 is widely accepted as a diagnostic marker of ovarian cancer and a number of other malignant conditions such as breast cancer, mesothelioma, non-Hodgkin lymphoma, pancreatic cancer, gastric cancer, and leiomyoma [83], with exemption of benign conditions including endometriosis, pregnancy, congestive heart failure, nephrotic syndrome, and fibrosis-associated disease including liver cirrhosis and pulmonary fibrosis [84,85,86,87,88]. Muc16 as well as Msln were identified as signature genes of aPFs isolated from mouse BDL liver [8], and Muc16 was shown to bind to Msln as the ligand, facilitating the TGFβ receptor-mediated activation cascade of PFs in cholestatic injury [14]. 

### 5.3. Thy1 (CD90)

Thy1 (CD90, cluster of differentiation 90) is a GPI-anchored cell surface protein with a single V-like immunoglobulin domain, originally discovered as a thymocyte antigen [89]. Thy1 is expressed in fibroblasts as well as neurons and hematopoietic cells [90,91,92]. The studies of bleomycin-induced lung fibrosis implicated Thy1 in inhibition of TGFβ1-dependent fibroproliferative responses in tissue fibroblasts. In accord, Thy1-deficient mice develop severe lung fibrosis with increased accumulation of myofibroblasts in comparison to the wild-type mice [93,94]. Thy1 is highly expressed in lung fibroblasts and the loss of Thy1 is associated with profibrotic and apoptosis-resistant phenotype of myofibroblasts [95,96].

Thy1 prevents TGFβ1-mediated fibroblast activation by modulating lipid raft-associated signaling via the Src-family kinase (SFK) and focal adhesion kinase (FAK) pathways, promoting fibroblast adhesion and limiting migration [97].Thy1 was also shown to inhibit extracellular activation of tissue-associated latent TGFβ1 via interaction with αν-β5 integrins at the cell surface [98], implicating the potential function of Thy1 as a mechano-sensor [99]. 

Human THY1 shares similar properties with mouse Thy1, and human soluble THY1 (hsTHY1) is crossreactive with mouse ligands, showing anti-fibrogenic effect. Binding of hsTHY1 to αvβ5 integrin was shown to prevent activation of latent TGFβ1 in lung fibroblasts [98]. Administration of hsTHY1 (but not hsTHY1-RLE with mutated integrin-binding RGD-like motif) was also shown to reverse TGFβ1-induced myofibroblast differentiation in a dose-dependent manner, suggesting that integrin-binding RGD motif of Thy1 is required for the reversibility of myofibroblast differentiation [100]. Our unpublished data showed that administration of hsTHY1 peptide (1 μg/g in PBS) attenuated cholestatic fibrosis in BDL-injured mice with reduced activation of aPFs as compared to mutant hsTHY1-RLE- or vehicle-treated mice. It is speculated that hsTHY1 blocks TBFβ1-TGFβRI signaling by disturbing Msln binding to TGFβRI.

## 6. Msln, Thy1, and Muc16 Signaling in Activation of Portal Fibroblasts

### 6.1. Msln and Muc16 Regulate TGFβ1-Inducible Activation of aPFs

Msln signaling is a key mediator of activation and proliferation of PFs and progression of cholestatic fibrosis. It has been demonstrated that Thy1 as well as Muc16 can bind to Msln, forming a Msln-Muc16-Thy1 complex to modulate profibrogenic effect in aPFs. 

In the experimental models of cholestatic fibrosis including BDL and Mdr2^−/−^ mice, Msln-deficiency exhibited a significant suppressive effect on the progression of liver fibrosis by ≈50% decrease in myofibroblast infiltration. Similarly, deletion of Muc16, the ligand of Msln, also attenuated liver fibrosis. Notably, deficiency in Msln or Muc16 in Mdr2^−/−^ mice reduced ductular reaction with significant downregulation of K19 and Sox9, which were shown to highly correlate with the reduced migration and proliferation of aPFs. Based on the physiological location of PFs in close proximity to bile ducts, it is suggested that proliferating aPFs and cholangiocytes strongly interact with each other in cholestatic injury [10,101]. In vitro analysis showed that TGFβ1-mediated phosphorylation of Smad2 and the expression of TGFβRI and αSMA associated with Col1a1 synthesis, as well as FGF-induced phosphorylation of Akt1 and the cell proliferation were downregulated in Msln^−/−^ aPFs as compared to Msln-expressing aPFs [14,17]. 

### 6.2. Ablation of Thy1 Exacerbates Cholestatic Fibrosis

In contrast to Msln^−/−^ mice with BDL- or Mdr2^−/−^-induced cholestatic fibrosis, Thy1^−/−^ mice developed more advanced fibrosis by ≈25%, which was associated with increased numbers of Col-GFP^+^CD34^+^ aPFs with significant upregulation of Col1a1, αSMA, TGFβRI, and Msln genes, supporting the inhibitory effect of Thy1 on activation of aPFs [14,17]. TGFβ1-induced Smad2 phosphorylation and αSMA protein expression were accelerated in Thy1^−/−^ aPFs as compared to wild-type aPFs, while those were suppressed in Msln^−/−^ and Muc16^−/−^ aPFs. 

Deletion of Thy1 in aPFs was associated with strong overexpression of Msln, indicating that Thy1 might negatively regulate the Msln signaling pathway. In turn, expression of TGFβRI protein was suppressed in Msln-deficient aPFs, suggesting that Msln might affect TGFβRI protein stability. The pro- and anti-fibrogenic responses of Msln and Thy1 in aPFs were offset by simultaneous deletion of Msln and Thy1. Opposing effects of Msln and Thy1 were completely diminished in Msln^−/−^Thy1^−/−^Mdr2^−/−^ mice to the levels observed in Mdr2^−/−^ mice, suggesting that Msln and Thy1 are key components of the same signaling pathway in aPFs [17].

### 6.3. Msln-Muc16-Thy1 Complex Regulates TGFβ/TGFβRI-Mediated Signaling in aPFs

**Figure 2:**
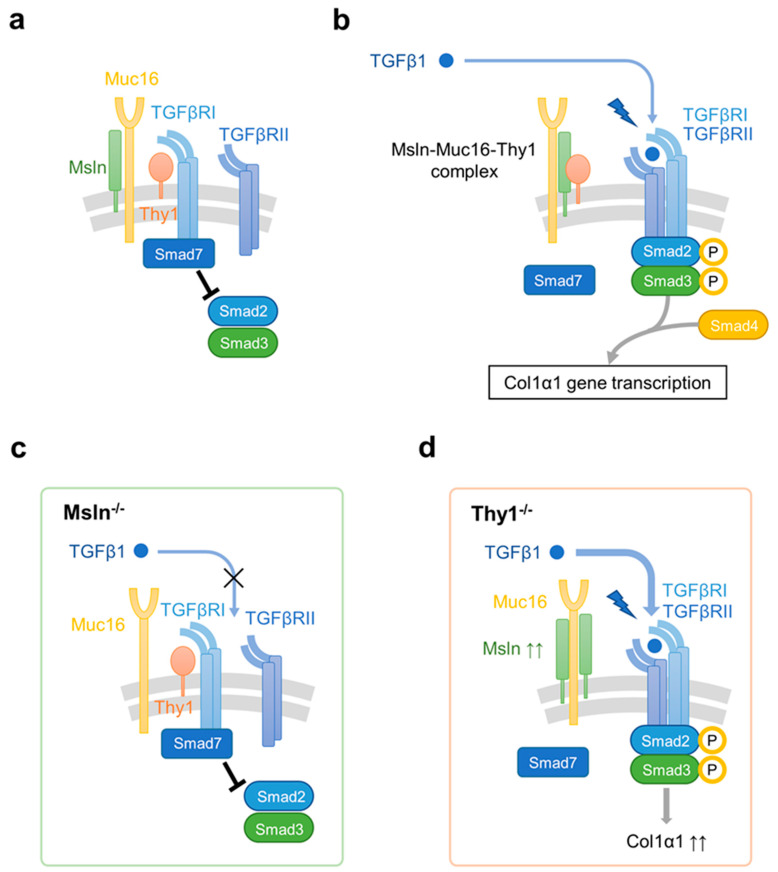
Proposed model of Msln-Muc16-Thy1 interaction in (**a**) resting wild-type PFs, and TGFβ1-stimulated (**b**) wild-type, (**c**) Msln^−/−^, and (**d**) Thy1^−/−^ aPFs. (**a**) Msln-Muc16 complex and Thy1-TGFβRI complex are formed in resting PFs. Binding of Thy1 to TGFβRI suppresses TGFβ1 signaling, while retaining Smad7 at the C-terminus of the TGFβRI. (**b**) In response to TGFβ1 signaling, Msln-Muc16 complex binds to Thy1, leading to dissociation of Thy1 from TGFβRI. TGFβ1 binding to TGFβRI and TGFβRII causes receptor crosslinking and binding of Smad2/3 to the receptors. Phosphorylated-Smad2/3 forms a complex with Smad4, and initiates transcription of target genes including Col1α1. (**c**) In Msln^−/−^ aPFs, increased affinity of Thy1 with TGFβRI hampers TGFβ1 binding to TGFβRI and TGFβRII, leading to attenuation of following phosphorylation of Smad2/3 and the downstream expression of Col1α1. (**d**) Thy1^−/−^ aPFs exhibit acceleration of Col1α1 synthesis in response to TGFβ1 stimulation due to the absence of Thy1 inhibition to TGFβRI, accompanied with increased phosphorylation of pSmad2/3 and reduced binding of Smad7 to TGFβRI.

A series of immunoprecipitations with specific antibodies for Msln, Muc16, Thy1, and TGFβRI revealed dynamic interaction between Msln-Muc16 and Msln-Thy1 in TGFβ1-stimulated aPFs. Under physiological (resting) conditions, Thy1 forms an inhibitory complex with TGFβRI, thereby preventing binding of TGFβ1 to the N-terminus of TGFβRI. Msln forms a strong complex with Muc16, transmitting intracellular signals from Msln-Muc16 complex. TGFβ1 signaling is further inhibited by Smad7 which is bound to the C-terminus of the TGFβRI and prevents phosphorylation of Smad2/3 at TGFβRI (Figure 2a). 

TGFβ1 stimulation enhances the binding affinity of Msln to Thy1, promoting dissociation of Thy1 from TGFβRI. Thus, formation of Msln-Muc16-Thy1 complex results in disruption of Thy1-TGFβRI interaction and removal of inhibitory Thy1 from TGFβRI, eventually allowing TGFβ1-TGFβRI-induced profibrogenic signaling to proceed. TGFβRI binds to TGFβRII, leading to dissociation of Smad7 from TGFβRI and subsequent binding of Smad2/3 to the C-terminus of TGFβRI. Phosphorylated Smad2/3 are released from TGFβRI into the cytoplasm where they form a complex with Smad4. p-Smad2/3-Smad4 are translocated to the nucleus, where they bind to the DNA and initiate transcription of the profibrogenic genes including type I collagen (Col1α1) [14,36,102,103,104] (Figure 2b).

### 6.4. Msln-Deficiency Suppresses TGFβ1-TGFβRI-Induced Activation of PFs

Deficiency of Msln results in a suppressive effect on TGFβ1-TGFβRI signaling in aPFs via the increased affinity of Thy1 binding to TGFβRI compared to that in wild-type aPFs, reflecting the enhanced inhibitory effect of Thy1 on TGFβ1 signaling. Under these circumstances, Smad7 is constitutively bound to the C-terminus of TGFβRI with decreased phosphorylation of Smad2/3, resulting in downregulation of fibrogenic genes (Figure 2c) [14,17].

### 6.5. Ablation of Thy1 Accelerates TGFβ1-TGFβRI-Induced Activation of PFs 

On the other hand, deletion of Thy1 in aPFs results in strong overexpression of Msln, suggesting that Thy1 is a critical regulator of Msln [17]. Thy1^−/−^ aPFs exhibit significantly increased synthesis of type 1 collagen in response to TGFβ1 stimulation, accompanied with increased phosphorylation of pSmad2/3 as well as upregulation of TGFβRI, while binding of Smad7 to TGFβRI is reduced (Figure 2d). It is suggested that ablation of Thy1 induces exacerbation of Msln signaling caused by the compensatory overexpression of Msln and its target genes. As both Thy1 and Msln are GPI-linked proteins, Thy1 might bind to other transmembrane signaling receptors (distinct from Muc16 with which Thy1 has minimal interaction [14]) or the lipid rafts proteins to mediate its function [105]. 

## 7. Common Fibrogenic Function of Msln in Tissue Fibroblasts across Organs

Tissue resident fibroblasts reside in the interstitium in a quiescent state and generally comprise a minor mesenchymal population in any normal tissues. Tissue fibroblasts can serve as major myofibroblast precursors in various organ fibrotic diseases, including not only liver but also lungs and kidneys [2,18,19,106,107,108,109]. aPFs and activated lung and kidney fibroblasts share similarities with expression of common markers including Msln, Thy1, Gremlin1, Calca, Upk1b, Fbln1, CD34, Asporin, Gpc3, Bnc1, and CD200 as well as markers of perivascular mesenchymal progenitor cells such as Gli1/2, Osr1, Mfap5, and Vit [110,111]. Based on the Msln expression in mesothelial components and fibroblast precursors, the common Msln-Thy1-mediated regulation of activation of tissue fibroblasts was analyzed using experimental lung and kidney fibrosis models [17].

In the lung, the most prevalent and pernicious form of fibrosis is idiopathic pulmonary fibrosis (IPF) [112,113]. Fibroblastic foci are one of the hallmarks of interstitial fibrosis in IPF, correlated to poor prognosis [114]. Although there is a great deal of controversy regarding the origins and heterogeneity of lung fibroblasts [115,116,117,118], lesional fibroblasts in IPF exhibit some similarity with liver myofibroblasts. Comparison of the pathways regulating myofibroblast differentiation in lung and liver, demonstrated that activated Portal Fibroblasts (aPFs) and Lung Fibroblasts (aLFs) share remarkable similarities, including expression of Collagen Type I, α-SMA, TGF-β1/2, and recently identified fibroblast markers Thy-1, Mesothelin (Msln) and Mucin16 (Muc16) [8]. Thy1 is silenced in lesional fibroblasts in IPF, and its expression in murine lung fibroblasts is decreased with progression of experimental bleomycin induced lung fibrosis [93,119]. Thy1 was identified as a fibrosis suppressor which prevents differentiation of lung fibroblasts into myofibroblasts (including Collagen Type I expression, cytokine and growth factor expression, migration, and cell survival). Upon activation, lung myofibroblasts upregulate TGFβ1-responsive genes (Activin and PAI-1) but downregulate expression of Thy1 [93,98,120,121,122]. Deletion of Thy1 in Thy1^−/−^ mice exacerbated bleomycin-induced lung fibrosis [123]. Thy1 modulates lipid raft-associated signaling via the Src-family kinase (SFK) and focal adhesion kinase (FAK) pathways, promoting fibroblast adhesion and limiting migration [97]. Recent data indicates that Thy1 can function as a mechanosensory [99], that inhibits extracellular activation of tissue-associated latent TGF-β1 via interaction with αν-β5 integrins at the cell surface [98].

Msln^−/−^ mice were protected by ≈50% from bleomycin-induced lung fibrosis as compared with wild-type mice, with reduced activation of Col-GFP^+^Thy1^+^ lung fibroblasts. On the other hand, Thy1^−/−^ mice showed more exacerbated lung fibrosis by 25% than wild-type mice, which was consistent with previous findings of the inhibitory effect of Thy1 on activation of lung fibroblasts [93]. 

Similar results were observed in mice with kidney fibrosis that was surgically induced by unilateral ureter obstruction (UUO). Kidney fibrosis was attenuated by ≈40% in Msln^−/−^ mice with reduced infiltration of Col-GFP^+^Thy1^+^ tubular fibroblasts, while aggravated by ≈25% in Thy1^−/−^ mice as compared with wild-type mice. Remarkably, only interstitial fibroblasts expressed Msln in fibrotic kidneys. Glomerular fibroblasts did not upregulate neither Msln nor Thy1. 

Unlike cholestatic fibrosis, Muc16^−/−^ mice were not protected from either lung or kidney fibrosis, suggesting that Muc16 may play a limited role in activation of lung and kidney fibroblasts. Muc16 was not expressed in fibrotic kidneys, indicating that Msln-Thy1 signaling might recruit another signaling molecule. In turn, Muc16 is expressed in the injured lungs. Deletion of Muc16 attenuates mortality in acute model of bleomycin injury in mice, but does not protect chronically injured mice from bleomycin-induced lung fibrosis. Whereas the functional significance of Msln-Muc16 complex for stimulation of TGFβ1-TGFβRI signaling was shown only in aPFs, formation of Thy1-Msln complex regulates TGFβ1-TGFβRI signaling not only in aPFs but also in lung fibroblasts. 

Analogous to the observations in the Mdr2^−/−^ cholestatic fibrosis model, Msln^−/−^Thy1^−/−^ mice with bleomycin-injured lung fibrosis or UUO-injured kidney fibrosis showed fibrotic phenotypes comparable with wild-type mice, supporting the consistency of opposing function of Msln and Thy1 in regulation of tissue fibroblasts activation [17]. 

## 8. Anti-Fibrotic Therapy Targeting Msln

Targeting Msln may be beneficial for treating parenchymal organ fibrosis including cholestatic fibrosis. Major classes of MSLN inhibitors in patients to block MSLN-MUC16-THY1 signaling pathway include anti-human MSLN Ab-immunotoxin, which causes death of human MSLN^+^ cells [72]; anti-MSLN blocking Abs, which can potentially suppress growth and proliferation of aPFs [124]; or recombinant hsTHY1, which neutralizes reactivity to αν-β5 integrins and binds to TGFβRI to prevent MSLN signaling [100]. 

### Anti-MSLN Ab-Immunotoxin Targeting MSLN^+^ aPFs

Immunotherapy-based strategy to target human MSLN-expressing cancer cells has been developed by Dr. Pastan and colleagues, pioneers in the field of cancer research. MSLN is a strong candidate for anti-cancer therapy with recombinant immunotoxins due to its distinctive expression in human malignancies [125]. Several generations of immunotoxins, such as SS1P and LMB100, were engineered by conjugation of anti-human MSLN SS1 Ab [72,79,126] to PE38 (truncated *Pseudomonas* exotoxin) [127], and successfully tested in clinical trials in patients with mesothelioma, ovarian cancer and pancreatic cancer [125,128,129,130]. Binding to MSLN, the entire recombinant immunotoxin molecule is internalized, leading to the release of PE38 into the cytosol and cellular apoptosis via inactivation of ADP-ribosylation/elongation factor 2 pathway [127,131].

Based on previous findings, in which genetic ablation of aPFs by using overexpression of Diphtheria Toxin α causes aPF apoptosis without causing structural liver damage and attenuates cholestatic fibrosis in BDL-injured mice [14], it is postulated that immunotoxin-based ablation of human aPFs may become a novel therapeutic strategy for PSC. It is demonstrated that SS1P and LMB100 immunotoxins can successfully kill human primary cultured aPFs in vitro as well as in vivo using xenograft mice, generated by adoptive transplantation of human primary aPFs into the livers of adult immunodeficient Rag2^−/−^γc^−/−^ mice [17], suggesting that immunotoxins can effectively cause apoptosis of human aPFs and attenuate cholestatic fibrosis. Generation of human aPF xenograft Rag2^−/−^γc^−/−^ mice serves as a useful tool to study in vivo the patient-specific responses of aPFs to MSLN inhibitors.

## 9. Conclusions

Investigation of the role of Msln, Muc16, and Thy1 in fibrosis and fibroblast activation across multiple organs, demonstrated that Msln^−/−^ mice are protected from cholestatic fibrosis caused by Mdr2 deficiency, bleomycin-induced lung fibrosis, and UUO-induced kidney fibrosis. We propose that Msln is a critical activator of tissue fibroblasts. Msln expression correlated with the stage of liver fibrosis in patients with PSC. Anti-MSLN Ab-immunotoxins, developed for cancer therapy, were used to target human MSLN^+^ aPFs in vitro and in vivo, and successfully killed human aPFs, suggesting that immunotherapy-based targeting of MSLN^+^ tissue fibroblasts might provide a new strategy for treatment of cholestatic fibrosis and fibrosis in other organs. It might not cure patients with cholestatic fibrosis, but can decrease fibroproliferative responses to bridge PSC patients to liver transplantation, or treatment of the etiological causes.

## Data Availability

Not applicable.
